# Late Entry to HIV Care Limits the Impact of Anti-Retroviral Therapy in the Netherlands

**DOI:** 10.1371/journal.pone.0001949

**Published:** 2008-04-09

**Authors:** Colette Smit, Timothy B. Hallett, Joep Lange, Geoff Garnett, Frank de Wolf

**Affiliations:** 1 HIV Monitoring Foundation Amsterdam, Amsterdam, Netherlands; 2 Imperial College London, London, United Kingdom; 3 Academic Medical Centre Amsterdam, Amsterdam, Netherlands; University of Cape Town, South Africa

## Abstract

**Objective:**

To explain differences in survival in the first three years of combination anti-retroviral therapy (cART) between HIV treatment centres in the Netherlands.

**Methodology/Principal Findings:**

We developed a mathematical simulation model, parameterised using data from the ATHENA cohort that describes patients entering care, being monitored and starting cART. Three scenarios were used to represent three treatment centres with widely varying mortality rates on cART that were differentiated by: (i) the distribution of CD4 counts of patients entering care; (ii) the age distribution of patients entering care; (iii) the average rate of monitoring the patients not on cART. At the level of the treatment centre, the fraction of Dutch MSM dying in the first three years of treatment ranged from 0% to 8%. The mathematical model captured the large variation in observed mortality between the three treatment centres. Manipulating the age-distribution of patients or the frequency of monitoring did not affect the model predictions. In contrast, when the same national average distribution of CD4 count at entry was used in all the scenarios, the variation in predicted mortality between all centres was diminished.

**Conclusions/Significance:**

Patients entering care with low CD4 counts appears to be the main source of variation in the mortality rates between Dutch treatment centres. Recruiting HIV-infected individuals to care earlier could lead to substantial improvements in cART outcomes. For example, if patients were to present with at least 400 CD4 cells/mm^3^, as they do already in some centres, then our model predicts that the mortality in the first three years of cART could be reduced by approximately 20%.

## Introduction

The Dutch Ministry of Health recognises 24 general and academic hospitals as HIV treatment centres in the Netherlands. The treatment centres and the HIV Monitoring Foundation (HMF) [1] are required to systematically monitor the quality of care they provide and patient outcomes. However, comparing different centres is not straight forward because indicators of good patient management and successful treatment (combination Anti-retroviral Therapy: cART) initiation are influenced by the characteristics of the patients that present as well as by the attributes of the centre itself.

Recent modelling studies have highlighted the theoretical importance of frequently monitoring CD4 changes over time and ensuring that cART programmes can recruit individuals early in infection [2]–[3]. This is because cART outcomes are strongly determined by the CD4 cell count when treatment is started [4]–[6]; if individuals do not enter care early, the possibility of initiating treatment when its effect is greatest is lost, and if individuals are not monitored frequently, it is less likely that treatment can be initiated at the right time [3]. It has also been shown that cART outcomes are linked to age, with older patients tending to have higher mortality rates in the first years of treatment.

Here, we explore different hypotheses to explain different observed mortality rates in Dutch treatment centres. The hypotheses are: (1) the quality of treatment administered varies between the treatment centres; (2) more frequent patient monitoring in some centres generates better patient survival; or (3) patients entering care earlier in some centres generates better patient survival. Our approach is to compare the observed mortality of patients in treatment centres with the predictions of a mathematical model that is parameterised to reflect the variation in the profile of patients entering care at each centre and the frequency of monitoring. Other aspects of the model and HIV disease are parameterised using data collected in the Netherlands in a national observation cohort [7].

## Results

### Observed Survival Outcomes

At the individual level, there was no indication that survival rates stratified by starting CD4 cell count varied between patients attending the different treatment centres (p = 0.42). For men starting treatment with CD4 cell counts less than 50, 11% die in the first three years; with CD4 cell counts 50–200, 2% die; with CD4 cell counts 200–250, 2% die; and with CD4 cell counts greater than 350, 0% die. Across all treatment centres, Dutch MSM who present with CD4 cell count <200 cell/mm^3^ have a 75% higher risk of dying in the first 3 years on cART compared to those who present with ≥350 cell/ mm^3^ (adjusted HR: 1.75; 95% CI: 1.01–3.01).

At the level of the treatment centre, the fraction of Dutch MSM dying in the first three years of treatment ranged from 0% to 8%. MSM treated in centre A had the same risk of dying as the national average (HR: 1.08; 0.51–2.29). In centre B, no men died in the first three years of treatment, so the risk of dying was estimated as zero. The risk of dying whilst on treatment in centre C was higher than the national average (HR: 2.22; 95% CI: 0.53–9.53). The higher risk of dying in this centre was highly statistically significant when all those treated were considered, but it does not reach statistical significance when only MSM are included in the analysis due the much smaller sample size (N = 36).

### Variation in Patients at Treatment Centres

**Figure 1:**
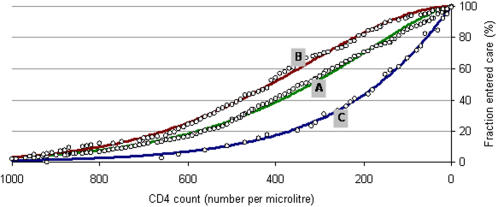
Distribution of CD4 count at presentation in three hospitals. Dots show data and lines show fitted Weibull curves with shape (α) and scale (β) parameters as follows: Hospital A α = 1.43 β = 414.46; Hospital B α = 1.83 β = 505.12; Hospital C α = 1.06 β = 236.45.

Figure 1 shows the cumulative distribution of CD4 counts among Dutch MSM presenting at the three treatment centres for the first time. The patients presenting at centre C typically had much lower CD4 counts than patients presenting at treatment centres A and B. The age of patients entering care was similar in all centres (mean age 32–34 years). Patients in care but not on treatment were observed, on average, every 5–6 months in centre A and B and every 3–4 months in centre C.

### Comparison with Model

**Figure 2:**
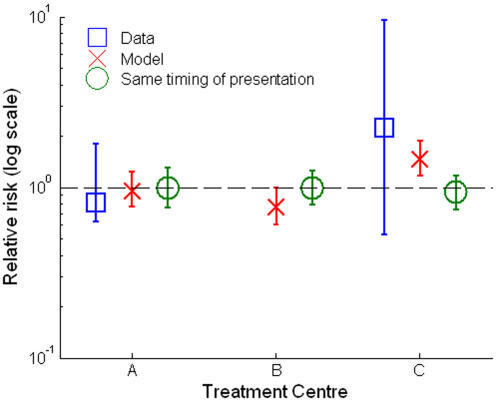
Observed and modeled risk of dying in first three-years of ART relative to national average. Errorbars show 95% confidence interval (data) or inter-quartile range from 500 simulations (model).

When the model is parameterised using the age distribution, frequency of monitoring and the distribution of CD4 cell counts at entry to care observed in each of the three treatment centres, the model captures the large variation in observed mortality on cART (Figure 2, cross). In particular, the model replicates the substantially greater risk of dying for patients in centre C (Figure 2).

If the rate of monitoring is increased in the model, mortality is slightly reduced in all centres by 5–10%. However, the model predicts large differences in overall mortality rates between the three treatment centres, even when the same monitoring frequency is used. Changing the age-distribution of patients in each treatment centre does not materially alter the overall survival rates on treatment. This is because the age of patients is not strongly independently linked to survival on cART, and the faster progression of older patients does not lead to substantially fewer of them starting treatment with the frequency of monitoring that is assumed here.

In contrast, when the same (national average) distribution of CD4 count at entry to care was used to simulate the three treatment centres (but with other parameters reflecting the differences between the centres), the model does not indicate an excess risk of dying at centre C and the variation between all centres is diminished (Figure 2, circles). This result is also found when lower and higher thresholds for starting treatment are assumed (Figure S1).

## Discussion

Large differences in survival on cART were observed in treatment centres in the Netherlands – the range of hazard rates during the first years on treatment varied between 0.34 and 3.28 over the 24 treatment centres. Based on recent modelling work [3], we hypothesised that this could be due to difference in standards of administering treatment at the centres, differences in the frequency of monitoring patients or variation in the profile of patients entering care (age and CD4 cell count when first presenting). When stratified by CD4 cell count at treatment initiation, survival in the subset of centres that we studied was very similar, indicating comparable standards of clinical management for the individual. The small differences in the frequency of clinical monitoring between the centres was not consistent with more frequent monitoring leading to better survival as we predicted. In the model, it was found that changes in the frequency of monitoring had little influence of the predicted mortality rates. The age of patients entering care was similar in all the treatment centres and did not explain differences in mortality rates. However, there was great variation in the CD4 cell count levels of patients entering care at the difference centres. For the model to qualitatively replicate the observed variation in mortality between the centres, including these different CD4 cell count distribution was both necessary and sufficient. We conclude that most of the observed variation in mortality between treatment centres can be explained by the timing of patients entering care.

Mathematical models have highlighted the importance of monitoring individuals from early in their infection [3]. In developed and developing countries alike [6], [8], [9], cART is more effective when started at higher CD4 levels, but not all patients enter care early enough to allow this [10]. Models also suggest that monitoring patients more frequently (e.g. every 6 months instead of every 12 months) before they need treatment can facilitate treatment being started at the right time. However, this effect was not strong for this population and it was outweighed by the larger influence of CD4 cell count at entry to care. This may be because the range of frequency of monitoring rates in the treatment centres was very narrow (mean interval between appointments in all centres ranged between 3 and 5 months) and the marginal benefit of monitoring more often is less when patients are already monitored that frequently. In our present analysis, the treatment centre with the highest frequency of monitoring (centre C) also had the highest mortality rate, and this may be due to clinicians scheduling more frequent appointments in response to the more advanced conditions among their patients due to the late entry to care.

We have used a novel approach to overcome the individual-level confounding effects that can undermine comparisons between health-care services. The complex, multi-faceted and non-linear nature of the relationship between patients, treatment centres and cART outcomes mean that statistical modelling may fail to completely adjust for all potentially confounding factors. In addition, statistical models can only provide phenomenological insights, whereas our modelling approach affords a mechanistic interpretation of the observed patterns. Our modelling work has provided independent evidence of the main factors determining – and limiting – survival outcomes on cART. We have also been able to test and verify modelling predictions with high-quality observational data.

The implication of our findings is that survival outcomes in many Dutch treatment centres would improve if patients entered care earlier in their HIV infection. Although most individuals are regularly tested for HIV and enter care when still healthy, still a substantial proportion of Dutch MSM (25%) presents after they have developed severe symptoms of immune-suppression [11]. In fact, Dutch MSM tend to present at the clinic with only marginally higher CD4 counts than men and women in West Africa [12].

Most of those entering care with high CD4 counts in centre B (the centre with the lowest mortality rates and patients entering the care earliest) were routinely tested for HIV as part of an ongoing cohort study. If Dutch MSM across the country were to enter care at the same time, then our model predicts that the mortality in the first three years of cART could be reduced by approximately 20%. Increased and early HIV testing is required for the full impact of cART to be realised in the Netherlands, because even the highest standards of clinical management cannot make up for the lost benefit of treating patients early.

## Methods

### Data: Treatment Outcomes and Profile of Patients in Dutch Treatment Centres

The risk of dying in the first three years on cART for all patients was assessed for each clinic using a multivariate Cox-proportional hazards model. The model was adjusted for variation in the following factors that have been shown to influence survival outcomes: gender; age; HIV risk group (categorised as: Men who have sex with men (MSM), heterosexual, injecting drug use or other/unknown (including blood contact and vertical transmission)); region of origin (categorised as: Netherlands, Western Europe and North America excluding the Netherlands, Sub Saharan Africa, Latin America/ Caribbean and other); calendar year of HIV diagnosis (categorised as: <2000, 2000–2002, 2003–2007); CD4 cell count at first presenting and, symptoms at first presentation at the centre (categorised as: symptoms present or not present). The risk of dying for each treatment centre was compared to the risk of dying in the total population using hazard ratios. The proportional hazard assumption was checked by examining the distribution of the Schoenfeld residuals.

On this basis, three HIV treatment centres in the Netherlands were selected for this study: centre A has approximately the same risk of dying as the national average (Hazard ratio (HR): 0.85; 95% confidence interval: 0.54–1.31); centre B has the lowest mortality rate (HR: 0.34; 0.12–0.98); and, centre C has the highest mortality rate (HR: 3.28; 1.93–5.56). The risk of dying in the other treatment centres varied between the risks of centres B and C.

To prevent differences in the socio-ethnicity status of patients interfering with the comparison with the model, the mortality rates in the three centres was then recalculated only for Dutch men that acquired HIV through sex with men (i.e. Dutch MSM) (n = 3946, 31% of the total dataset). Among these men, 21% initiated cART before 1997 and 79% were initiated between 1997 and 2007.

For each of these three treatment centres, the following distributions were found for the Dutch MSM entering care: (1) the age-distribution (in five-year groups); (2) the average rate of clinic visits for the patients being monitored for the need for cART (i.e. those not yet on treatment); and, (3) the distribution of CD4 cell counts when first entering care. These data are used to parameterise the mathematical model scenarios corresponding to these centres.

### Data: Natural History of HIV Infection among Dutch Men

Data from the ATHENA national observational cohort [13] were used to estimate the common biological parameters describing the natural history of HIV infection in this population. That is, we assume that there are no differences in the clinical course of infection between the men attending the different treatment centres (this assumption is tested, see Results). The model (described in more detail below) represents the course of infection by simulating the decline in CD4 cell counts from an initial starting value immediately after seroconversion. We used square-root transformed values of the CD4 cell counts. The distribution of the CD4 cell count 6 months after seroconversion for Dutch MSM was estimated in a random effects model to have a mean value of 22.5, normally distributed with standard deviation 0.45, for men aged less than 35; and mean 21.2 and standard deviation 0.45 for men aged 35 years and older. The rate of decline in CD4 cell count after the first 6 months of infection was assumed to be linear on the square-root scale, which is in keeping with other analyses [14] [15] [16] and theoretical [17] and clinical [18] observations. For men aged less than 35 years, the rate of decline is 1.86 (Normally distributed with standard deviation 0.17); for men aged 35 years or more, the rate is 2.10 (standard deviation 0.17).

Three-year survival rates on ART, stratified by CD4 cell count when treatment is initiated, were estimated using ATHENA data, pooled across all the treatment centres.

### Mathematical model

A mathematical model representing patients entering care, being monitored for the need to start treatment and treatment outcomes was developed. The model has been described previously [3] but the key features are described again here.

The model represents a cohort of individuals infected with HIV at the same time. Each individual is assigned particular characteristics (drawn randomly from the parametric distributions estimated as described above), including their age at seroconversion, the CD4 cell count after seroconversion, the rate of CD4 decline and the CD4 level at which they enter care in a treatment centre.

Once in care, the need for cART is assessed. A CD4 cell count is taken, but the measured value incorporates some measurement error (uniformly distributed with mean zero) reflecting short-term physiological variability and technical laboratory factors [19] [20]. If the CD4 cell count measurement is less than 250 cells per cubic millimetre, then treatment is started. This threshold is based on the median CD4 count at time cART initiation among Dutch MSM in the Netherlands (median: 210 IQR: 100–320) and a sensitivity analysis is conducted to check the influence of this assumption.

Whether or not that individual survives on cART for three-years is determined probabilistically based on the estimated chance of survival for individuals with that CD4 cell count. If cART is not needed when the patient first enters care, the need for cART is assessed again at another ‘appointment’ scheduled after a set interval. After the CD4 cell count level reaches 50 and if the patient has not started cART, is it assumed that survival is exponentially distributed with mean 6 months.

The model scenarios representing the three treatment centres were differentiated by the following three ‘treatment centre’ parameters: the age distribution of patients entering care, the average rate of clinic visits of the patients not on cART and the distribution of CD4 counts of patients first entering care. We investigated: (1) whether the chance of individuals surviving on cART varied between treatment centres when patients are stratified by the initial CD4 cell count; (2) whether the model could reproduce the observed variation in mortality between the three treatment centres when parameterised in this way; and, (3) the relative influence of these treatment centre parameters on the predicted level of mortality on treatment.

## Supporting Information

Figure S1 Sensitivity analysis. The analysis shown in Figure 2 in the main text is repeated using alternative assumptions about the CD4 cell count level at which treatment is initiated: (A) at 200 cell per microlitre or less; (B) at 350 cells per microlitre or less. The blue squares show the observed data; the red cross show the model simulation with the parameters chosen to reflect conditions in each treatment centre; the green circles show the model simulation when the parameters are chosen to reflect conditions in each treatment centre, with the exception that the distribution of CD4 cell count among patients entering care is the same in all centres.
(0.03 MB TIF)
